# Photodynamic Therapy of Lung Cancer With Bronchial Artery Infusion of Photofrin

**DOI:** 10.1155/DTE.2.203

**Published:** 1996

**Authors:** Tetsuya Okunaka, Harubumi Kato, Chimori Konaka, Kinya Furukawa, Masahiko Harada, Yutaka Yamamoto

**Affiliations:** Department of Surgery Tokyo Medical College 6-7-1, Nishishinjuku Shinjuku-ku Tokyo 160 Japan

## Abstract

Photodynamic therapy (PDT) utilizing Photofrin is proving to be effective for the treatment of early stage lung cancer. However, wider clinical applications of Photofrin as a photosensitizer for various cancers are hampered by potentially serious and prolonged skin photosensitivity. To prevent these side effects and reduce the hospitalization period, we recently gave reduced doses of Photofrin by bronchial arterial infusion. Five patients with endoscopically evaluated minimally invasive carcinoma of the lung were given 0.7 mg/kg of Photofrin by bronchial arterial infusion 48 hr before PDT. Complete remission was obtained in all 5 cases and no case showed skin photosensitivity when exposed to sunlight under careful surveillance at one week after PDT.


*Diagnostic and Therapeutic Endoscopy*, 1996, Vol. 2, pp. 203–206
Reprints available directly from the publisher
Photocopying permitted by license only

© 1996 OPA (Overseas Publishers Association)
Amsterdam B. V. Published in The Netherlands
by Harwood Academic Publishers GmbH
Printed in Singapore

### Photodynamic Therapy of Lung Cancer with Bronchial Artery Infusion of Photofrin

TETSUYA OKUNAKA, HARUBUMI KATO, CHIMORI KONAKA, KINYA FURUKAWA, MASAHIKO HARADA, and YUTAKA YAMAMOTO

*Department of Surgery, Tokyo Medical College, Tokyo, Japan*

*(Received March 15, 1995; in final form December 25, 1995)*

Photodynamic therapy (PDT) utilizing Photofrin is proving to be effective for the treatment of early stage lung cancer. However, wider clinical applications of Photofrin as a photosensitizer for various cancers are hampered by potentially serious and prolonged skin photosensitivity. To prevent these side effects and reduce the hospitalization period, we recently gave reduced doses of Photofrin by bronchial arterial infusion. Five patients with endoscopically evaluated minimally invasive carcinoma of the lung were given 0.7 mg/kg of Photofrin by bronchial arterial infusion 48 hr before PDT. Complete remission was obtained in all 5 cases and no case showed skin photosensitivity when exposed to sunlight under careful surveillance at one week after PDT.

**KEY WORDS:** Photodynamic therapy, lung cancer, bronchial arterial infusion

### INTRODUCTION

Photodynamic therapy (PDT) using hematoporphyrin derivative as an effective modality in the management of cancer is currently receiving considerable attention (1–3). The photosensitizer preparation most commonly used is a complex mixture of porphyrins termed hematoporphyrin derivative (HpD) or Photofrin, a partially purified preparation of HpD, which received government approval on October 1994 in Japan. In spite of excellent results in clinical studies, especially in early-stage lung cancer, wider clinical applications of PDT using HpD are hampered by the skin photosensitivity it induces (4,5). Photofrin has routinely been given intravenously at a dosage of 2.0 mg/kg body weight since the report of Dougherty et al. (6); however, patients receiving Photofrin must avoid sunlight for a minimum of 30 days to avoid sunburn (4).

On the other hand, regional infusion of anticancer agents into the malignant lesion via a transarterial route could minimize fast deactivation while increasing the anticancer effect (7). Bronchial artery infusion (BAI) is known to achieve high concentrations of anticancer drug in lung cancer tissue (8). To reduce skin photosensitivity and to increase local photodynamic effects, a clinical study of low-dose Photofrin given by BAI was performed.

Address for correspondence: Dr. T. Okunaka, Department of Surgery, Tokyo Medical College, 6-7-1, Nishishinjuku, Shinjuku-ku, Tokyo 160, Japan. Tel. 813-3342-6111 (ext. 5071); Fax: 813-3349-0326.

### MATERIALS AND METHODS

### Patient Selection

The patients and their histories are outlined in Table 1. Five patients with endoscopically evaluated early-stage carcinoma of the lung and two with advanced carcinoma were treated. Histologically, all cases were squamous cell carcinoma. All 7 patients were men with a mean age of 73 years (range 60 to 81 years).

### Procedure

The photosensitizer used was porfimer sodium (Photofrin, Lederle Japan Inc., Tokyo, Japan). One or more arteries feeding the tumor were identified angiographically before infusion of Photofrin. After catheterization of the target artery, a single dose of Photofrin (0.7 mg/kg body weight) was slowly infused into the bronchial artery followed by endoscopic PDT performed with topical anesthesia approximately 48 hr later. The new laser light delivery system consists of an excimer laser, which emits a pulse laser beam coupled to a dye laser (Hamamatsu Photonics Ltd,

**Table 1** Results of BAI-PDT

| Patient | Age (yr) | Sex | Location of Tumor | Histologic Type* | Stage | Result | Skin Photo-sensitivity |
|---|---|---|---|---|---|---|---|
| 1 | 81 | M | Right $B^6$ orifice | Sq | I (T1N0M0) | CR | (–) |
| 2 | 77 | M | Left $B^6$ | Sq | I (T2N0M0) | CR | (–) |
| 3 | 77 | M | Right $B^7$ orifice | Sq | I (T1N0M0) | CR | (–) |
| 4 | 70 | M | Left $B^{1+2}$ | Sq | I (T1N0M0) | CR | Grade 1 |
| 5 | 60 | M | Right main bronchus | Sq | I (T2N0M0) | CR | (–) |
| 6 | 79 | M | Left 2nd carina | Sq | IIIA (T2N2M0) | PR | (–) |
| 7 | 68 | M | Carina | Sq | IV (T4N2M1) | PR | (–) |

*Sq, squamous cell carcinoma.

Hamamatsu, Japan) (9). The excimer laser uses a gas mixture containing 0.9% Xe, 0.1% HCl, and 99% helium at 2 atm pressure. The optimal performance of this laser is obtained at 30 mJ/pulse with one-half peak power × 10.9 nsec at 308 nm. The XeCl excimer laser (308 nm) can be coupled to a pump system, which contains 2 M Rhodamine B dye in ethanol to convert the beam to 630 nm. The beams from the excimer-dye lasers were focused onto 400 μm fused silica fibers (Moritex Ltd, Nagoya, Japan), the tips of which were fitted with microlens to improve the homogeneity of light distribution throughout the treatment field. The final circular area of illumination was 1 $cm^2$. Illumination times ranged from 10–40 mins, giving energy densities of 100 to 200 $J/cm^2$.

The tumor response to PDT was evaluated endoscopically, roentgenographically, cytologically, and histologically 1 month after treatment. Tumor response was classified into three categories: complete remission (CR), partial remission (PR), and no change (NC). CR was defined as the complete absence of tumor endoscopically as well as both cytologically and histologically. PR was defined as a 50% or more reduction in tumor volume estimated macroscopically or histologically, and NC was defined as a reduction in tumor volume of less than 50%.

Skin photosensitivity was tested within 72 hr after Photofrin administration (10). Testing was done on the lower back. Test rectangles 1.5 by 2.5 cm were exposed to visible light (400 to 750 nm) to cover the entire visible absorption spectrum of Photofrin. The light source for testing was a 500 W projector lamp with a filter to remove infrared light. The test light power density was 30 $J/cm^2$. Skin reaction was evaluated 24 hr after light exposure. This was graded as 0, 1+, 2+, or 3+. Grade 0 was defined as a normal skin without any skin reaction; a 1+ reaction indicated minimum erythema and/or mild redness. Moderate redness with edema but no blistering or necrosis was represented by 2+. A 3+ reaction indicated redness, edema, and blistering. Patients with no skin reaction to skin photosensitivity tests, were allowed to be exposed to sunlight under careful surveillance at 1 week after PDT (10).

### RESULTS

Table 1 shows the summary of results of BAI-PDT. Complete remission was obtained in all five patients with early-stage lung cancers, and partial remission was obtained in the two patients with advanced disease. Only one patient, (patient 4) showed mild, grade 1 photosensitivity. There was no evidence of skin photosensitivity in the other six patients.

### Case Report

In this 81-year-old man (patient 1), lung cancer was initially diagnosed based on positive sputum cytologic findings during a mass screening program for individuals at risk for lung cancer. All roentgenographic examinations were negative. The tumor was nodular, 5 by 5 mm in size and located in the right $B^6$ (Fig. 1A). It was treated by BAI-PDT using an excimer-dye laser at 100 $J/cm^2$. The tumor disappeared completely and a CR was obtained (Fig. 1B). Now, he is apparently disease-free 18 months after BAI-PDT. Skin photosensitivity test revealed grade 1 skin photosensitivity. No other side effects were recognized.

### DISCUSSION

More than 3000 patients with a wide variety of malignancies have been treated by PDT (11). In our institution, 211 patients with central type lung cancers including 66 patients of endoscopically evaluated early-stage lesions have been treated with PDT since 1980, and a CR rate of 66.7% was achieved for early-stage lesions treated only with PDT (12). In spite of these excellent results, PDT has not yet been generally been accepted as a standard cancer therapy, such as radiation or chemotherapy. One of the reasons is its side effect, skin photosensitivity. Among patients who did not heed recommendations to avoid sunlight and use sunscreens, many (90%) had skin photosensitivity due to the retention of appreciable amounts of photo-

A

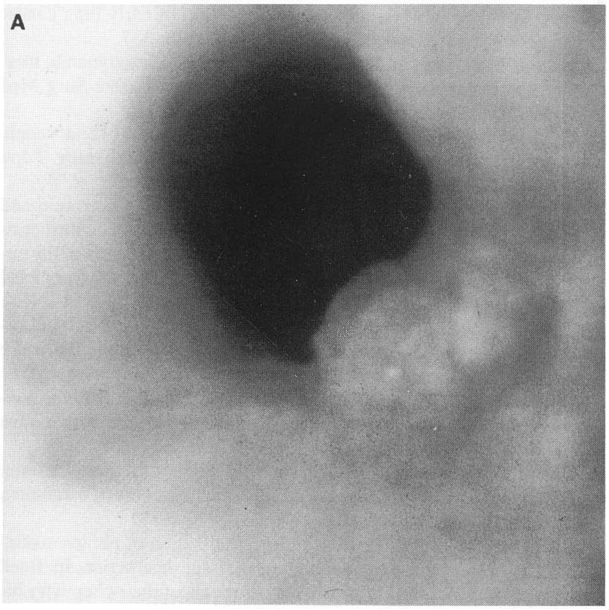

B

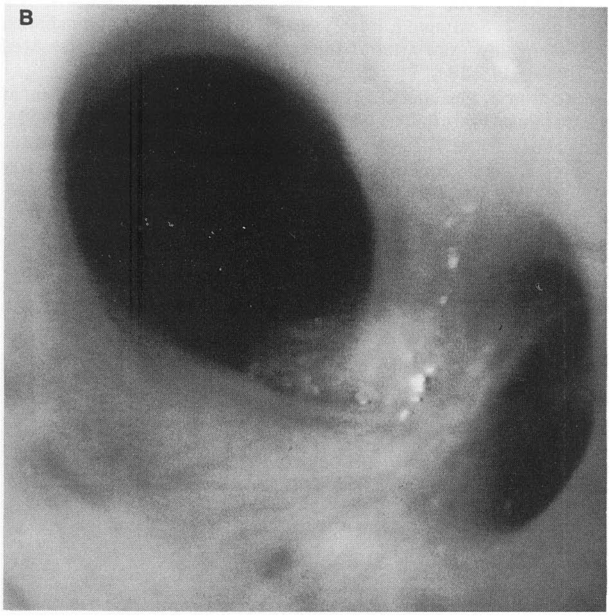

**Figure 1** Patient 1: A, nodular tumor at the right $B^6$, 5 by 5 mm in size; B, shows the same site 2 months after BAI-PDT.

sensitizers in the skin. This skin reaction is potentially so severe that patients must be warned to avoid exposure to direct sunlight for several weeks (4).

Preliminary studies in animals suggest that low doses (0.5 mg/kg) of Photofrin eliminate skin photosensitivity (13); in addition, Lam et al. (10) indicated that 0.25 mg/kg of Photofrin is enough to diagnose early-stage lung cancer without any skin photosensitivity (10). On the other hand, the selective infusion yields a higher uptake of drug in tumor tissues. The platinum concentration in resected tumor specimens has been reported to be 2 to 4.5 times higher than in normal tissue, and clinical and histologic studies have shown a recognizable effect after BAI (8,14,15). The Seldinger method is now used for puncture. Skill and experience should enable the endoscopist to successfully perform this even in patients with arteriosclerosis and anatomic abnormalities (8,16). Adverse reactions—intra-arterial infusion, infection and thrombosis around the insertion site of catheters, hemorrhage due to catheter damage, and intimal damage during insertion of catheter—may occur; however, serious complications such as paralysis were not reported (16). Therefore, to increase the concentration of drug in the tumor and decrease systemic distribution in the rest of the body, it seems logical to infuse the drug directly into the tumor-feeding bronchial artery.

Unfortunately, the photosensitizer pharmacokinetics and distribution in man are still unknown (17); however, an experimental study in dogs revealed that intra-arterial injection of Photofrin achieved higher Photofrin accumulation than intravenous injection (18). Kostron et al. (19) reported the effectiveness of PDT with intra-arterial injection of Photofrin for brain tumors.

Lung cancer is a difficult disease to treat curatively because of its systemic nature, development in older persons, and multiple primary lesions. Surgery has proved to be the best curative modality so far. However, in the authors' institution, the overall 5-year survival rate is still only 18% during the past 42 years. As opposed to this, in the early-stage central-type lung cancer with the tumor invasion of 1 cm or less, we obtained a 5-year survival rate of 100% (20). To control the lung cancer death rate, mass surveys for the detection of early-stage lung cancer, have been used in the elderly with annual chest x-ray and sputum cytology for the past 7 years. However, surgical treatment is limited in older persons, and there is also the problem of multiple lung cancers in this aged group.

In this study complete remission was obtained in all patients with minimally invasive lung cancers treated by BAI-PDT. No evidence of significant skin photosensitivity was obtained in all patients when the low dose of 0.7 mg/kg. Photofrin was given by BAI. These results may suggest that BAI-PDT holds promise for clinical application. Precise dosimetry yet remains to be established, and safe exposure to an unlimited amount of visible light in the natural environment has not been established in patients receiving Photofrin. Lam et al. (10) suggested that there was a threshold for skin photosensitivity reaction between 0.25 and 2 mg/kg Photofrin; however, further work on dosemetry remains to be performed.

### ACKNOWLEDGMENT

This work was supported in part by grants from the Ministry of Health and Welfare and the Ministry of Industry and Trade, Japan. The authors wish to thank Professor J. P. Barron of Tokyo Medical College for his review of the manuscript.

### REFERENCES

1. Hayata Y, Kato H, Konaka C, et al. Hematoporphyrin derivative and laser photoradiation in the treatment of lung cancer. Chest 1982;81:269–277.
2. Okunaka T, Kato H, Konaka C, et al. Photodynamic therapy of esophageal carcinoma. Surg Endosc 1990;4:150–153.
3. Hayata Y, Dougherty TY, eds. Lasers and hematoporphyrin derivative in cancer. Tokyo: Igaku-Shoin, 1983.
4. Razime N, Balchum OJ, Profio AF, et al. Skin photosensitivity; duration and intensity following intravenous HpD and DHE. Photochem Photobiol 1987;46:925–928.
5. Wooten RS, Smith KS, Ahlquest DA, et al. Prospective study of cutaneous phototoxicity after systemic hematoporphyrin derivative. Lasers Surg Med 1988;8:294–300.
6. Dougherty TJ, Laurence G, Kaufman JH, et al. Photoradiation in the treatment of recurrent breast carcinoma. J Natl Cancer Inst 1979;62:231–237.
7. Kahn PC, Paul RE, Rheinlander HF. Selective bronchial arteriography and intra-arterial chemotherapy in carcinoma of the lung. J Thorac Cardiovasc Surg 1965;50:640–645.
8. Masuda H, Ogata T, Kikuchi K, et al. Bronchial arterial infusion of cisplatin for lung cancer and platinum concentration in the tumor. Lung Cancer 1988;28:297–302.
9. Yamamoto H, Kato H, Okunaka T, et al. Photodynamic therapy with the excimer dye laser in the treatment of respiratory tract malignancies. Lasers Life Sci 1991;4:125–133.
10. Lam S, Palcic B, McLean D, et al. Detection of early lung cancer using low dose Photofrin II. Chest 1990;97:333–337.
11. Marcus SL, Dugan M. Global status of clinical photodynamic therapy: the registration process for a new therapy. Lasers Surg Med 1992;12:318–324.
12. Okunaka T, Konaka C, Tsutsui H, et al. Present status of endoscopic treatment of lung cancer: the role of photodynemic therapy. J Jpn Soc Bronchol 1994;16:712–722.
13. Mang TS, Potter WR, Dougherty TJ. Fluorescence detection and HPLC characterization of intratumoral porphyrin in occult metastasis of the lymph nodes following injection of low levels of the purified component of hematoporphyrin derivative. Lasers Surg Med 1988;8:145–146.
14. Ten GJ, Chai XL, Gao GR, et al. Intraarterial digital subtraction angiography in bronchogenic carcinoma treated with bronchial artery infusion. Eur J Radiol 1991;12:91–94.
15. Uchiyama M, Kobayashi H, Nakajo M, et al. Treatment of lung cancer with bronchial artery infusion of cisplatin and intravenous sodium thiosulfate rescue. Acta Oncol 1988;27:57–61.
16. Ogata T, Suemasu K, Yoneyama T, et al. Study of regional arterial infusion of anticancer agents as an adjuvant to surgery in carcinoma of the lung. Jpn J Cancer Clin 1977;23:1085–1089.
17. Henderson BW, Bellnier DA. Tissue localization of photosensitizers and the mechanism of photodynamic tissue destruction. In: Bock G, Harnett S, eds. Photosensitizing compounds: their chemistry biology and clinical use. Chichester: John Wiley and Sons, 1989:112–130.
18. Andou T, Sekimoto I, Hoshiyama N, et al. Study of photoradiation therapy by intra-arterial injection of HpD. J Jpn Laser Med 1987;7:143–144.
19. Kostron H, Plangger C, Fritsch E, et al. Photodynamic treatment of malignant brain tumor. Wein Klin Wochenschr 1990;102:531–535.
20. Kato H, Horai T, Furuse K, et al. Photodynamic therapy for cancers: a clinical trial of porfimer sodium in Japan. Jpn J Cancer Res 1993;84:1209–1214.

